# Rapid Detection of Genotype II African Swine Fever Virus Using CRISPR Cas13a-Based Lateral Flow Strip

**DOI:** 10.3390/v14020179

**Published:** 2022-01-18

**Authors:** Ning Wei, Bohan Zheng, Junjun Niu, Tao Chen, Jing Ye, Youhui Si, Shengbo Cao

**Affiliations:** 1 State Key Laboratory of Agricultural Microbiology, Huazhong Agricultural University, Wuhan 430070, China; weining@webmail.hzau.edu.cn (N.W.); zhengbohan@webmail.hzau.edu.cn (B.Z.); njj@webmail.hzau.edu.cn (J.N.); ct@webmail.hzau.edu.cn (T.C.); yej@mail.hzau.edu.cn (J.Y.); 2 Laboratory of Animal Virology, College of Veterinary Medicine, Huazhong Agricultural University, Wuhan 430070, China; 3 Key Laboratory of Preventive Veterinary Medicine in Hubei Province, The Cooperative Innovation Center for Sustainable Pig Production, Wuhan 430070, China

**Keywords:** CRISPR/Cas13a, RAA, lateral flow strip, African swine fever, detection

## Abstract

The African swine fever virus (ASFV) is a dsDNA virus that can cause serious, highly infectious, and fatal diseases in wild boars and domestic pigs. The ASFV has brought enormous economic loss to many countries, and no effective vaccine or treatment for the ASFV is currently available. Therefore, the on-site rapid and accurate detection of the ASFV is key to the timely implementation of control. The RNA-guided, RNA-targeting CRISPR effector CRISPR-associated 13 (Cas13a; previously known as C2c2) exhibits a “collateral effect” of promiscuous RNase activity upon the target recognition. The collateral cleavage activity of LwCas13a is activated to degrade the non-targeted RNA, when the crRNA of LwCas13a binds to the target RNA. In this study, we developed a rapid and sensitive ASFV detection method based on the collateral cleavage activity of LwCas13a, which combines recombinase-aided amplification (RAA) and a lateral flow strip (named CRISPR/Cas13a-LFD). The method was an isothermal detection at 37 °C, and the detection can be used for visual readout. The detection limit of the CRISPR/Cas13a-LFD was 10^1^ copies/µL of p72 gene per reaction, and the detection process can be completed within an hour. The assay showed no cross-reactivity to eight other swine viruses, including classical swine fever virus (CSFV), and has a 100% coincidence rate with real-time PCR detection of the ASFV in 83 clinical samples. Overall, this method is sensitive, specific, and practicable onsite for the ASFV detection, showing a great application potential for monitoring the ASFV in the field.

## 1. Introduction

African swine fever (ASF) is a severe infectious disease of swine caused by the African swine fever virus (ASFV), which can have a devastating impact on the pig industry [1,2]. The ASFV is a large double-stranded DNA virus belonging to the family *Asfarviridae* [3]. The viral genome comprises between 170 and 190 kilobases that encode more than 150 ORFs (Open Reading Frame) and approximately 165 viral proteins [4,5]. The viral capsid protein p72, encoded by the B646L gene, is highly conserved and well characterized, making it a widely used target for both nucleic acid detection and phylogenetic analysis [6,7]. ASF has been spreading rapidly in China ever since its emergence in 2018, severely destroying the Chinese swine industry and food safety [8]. ASF affects domesticated and wild pigs of all breeds and ages, with a high mortality rate of nearly 100%. Normally, ASF presents with high fever, cyanosis of the skin, and severe bleeding in the lymph nodes [9]. There is no effective vaccine or treatment against ASF yet, so the prevention and control of African swine fever are still dominated by strict biological safety measures and the elimination of positive pigs [10,11,12]. Therefore, the establishment of rapid and accurate on-site diagnostic methods is of great significance for the scientific and effective prevention and control of the disease.

The existing ASFV detection gold standard recommended by the World Organization for Animal Health (OIE) is virus isolation, which is time-consuming and labor-intensive, and virus isolation is not a method applicable to disease prevention and control [13]. The diagnosis of the ASFV normally relies on both antibody and viral genome detection. When using viral antibody detection, most serological detection assays rely on the ELISA format, as it is simple and cost-effective. However, it lacks sensitivity in subacute or early-stage infections, because antibody induction takes a comparatively long time following viral infection [14,15]. Hence, the tools for detecting ASFV genomic DNA, such as polymerase chain reaction (PCR) and real-time PCR, have become popular because of their sensitivity and specificity for ASF diagnosis [16,17,18]. However, a major bottleneck of these techniques is the need for well-trained professional operations and sophisticated instruments, thus hindering their large-scale application in the field.

Microbial clustered regularly interspaced short palindromic repeats (CRISPR) and CRISPR-associated (Cas) systems recognize and cleave specific nucleic acid sequences (namely through cis-cleavage). Cas13a, also known as C2c2, functions as an RNA-guided RNA-targeting endonuclease. In addition, CRISPR-Cas13a can detect the presence of an RNA target by CRISPR RNA (crRNA) and the collateral cleavage activity of Cas13a [19,20,21]. SHERLOCK, a Cas13a-based molecular detection platform, was established by combining the amplification of recombinase polymerase amplification (RPA), with T7 transcription of amplified DNA to RNA and the collateral effect of CRISPR-Cas13a [22]. CRISPR-Cas13a-based detection has been successfully applied to detect severe acute respiratory syndrome coronavirus 2 (SARS-CoV-2), Ebola virus, dengue virus (DENV), Zika virus (ZIKV), and other molecules [23,24,25,26].

To construct a more convenient method for detecting the ASFV, we developed a detection system by combining CRISPR/Cas13a, recombinase-aided amplification (RAA), and a lateral flow strip in this study. We designed a specific primer and probe combination for the conservative sequence of the p72 gene, optimized the sensitivity and finally tested its specificity using clinical samples. We believe this method can become an alternative approach to on-site ASFV detection and will help the timely monitoring and rapid formulation of ASF control strategies.

## 2. Materials and Methods

### 2.1. Viral Nucleic Acid Samples

The number and type of samples tested in this study were summarized below. Briefly, the field samples including 33 blood, 25 nasopharyngeal swabs, 7 spleens, 5 liver, 9 lungs, and 4 kidneys were individually collected and provided by the Detection Laboratory of Animal Disease Diagnostic Center, Huazhong Agricultural University, Hubei, China. Viral genomic DNA was extracted by using a Quick-DNA Viral kit (TIANGEN, Beijing, China).

### 2.2. Reagents and Instruments

A HiScribe T7 Quick High Yield RNA Synthesis kit was purchased from New England Biolabs (Ipswich, MA, USA). A DNA Fragment Purification Kit was obtained from TIANGEN Biotech Co., Ltd. (Beijing, China). T4 DNA Ligase, RNase inhibitor, *EcoRI*, and *BamHI* endonucleases were obtained from TAKARA (Dalian, China). Agencourt RNAClean XP was purchased from Beckman. An RAA kit, a lateral flow strip, and LwCas13a were purchased from Anhui Microanaly Genetech Co., Ltd. (Hefei, China).

### 2.3. RAA Primer Design and crRNA Preparation

Seventeen p72 sequences, covering genotypes I, II, III, IV, V, and X, were selected as reference sequences for the primer design. Nucleotide sequences of ASFV p72 genes were aligned to identify the conserved regions. The RAA primers were selected in the conserved nucleotide region of the p72 gene according to the RAA primer’s design requirements. The T7 promoter sequence (GAAATTAATACGACTCACTA TAGGG) was appended to the 5′-end of the RAA forward primer. Five sets of primers were designed. The primers were synthesized by Sangon Biotech (Table 1).

Five Cas13a crRNAs targeting the RAA-amplified products of p72 were designed. For the crRNA preparation, the DNA templates of crRNA were appended with a T7 promoter sequence and then were synthesized as primers by Sangon Biotech (Table 2). Two primers were annealed to a double-stranded DNA using an annealing buffer. The double-stranded DNA was purified by gel extraction. According to the instructions of the HiScribe T7 Quick High Yield RNA Synthesis Kit (New England Biolabs), the double-stranded DNA was transcribed to crRNA by incubating overnight at 37 °C. According to the manufacturer’s instructions, the crRNA was purified using Agencourt RNAClean XP (Beckman). The crRNA was stored at −80 °C.

### 2.4. Standard Plasmid Preparation

The p72 gene (GenBank: MK333180.1) fragment (Supplementary File S1) of the ASFV was synthesized by Sangon Biotech (Shanghai, China), then subcloned into pUC57 to generate a pUC57-p72 plasmid, which was used as the detection target unless other illustrated. The resulting plasmid was transformed into *E. coli* (*Escherichia* *coli*) DH-5α and verified by restriction enzyme analysis and DNA sequencing.

### 2.5. Evaluation and Optimization of the RAA Reaction

The RAA reactions were conducted using the RAA kit according to the manufacturer’s instructions. Briefly, a 50 μL reaction assembled with 25 μL buffer A, 13.5 μL nuclease-free water, 4 μL DNA sample, 1 μL RAA polymerase, 2 μL p72-RAA-F (forward primer, 10 μM), 2 μL p72-RAA-R (reverse primer, 10 μM), and 2.5 μL magnesium acetate was incubated at 39 °C for 30 min. Different primers were tested to select the best-performing primer pair. Subsequently, the RAA reaction products were transferred to the CRISPR/Cas13a cleavage assay.

### 2.6. CRISPR/Cas13a-LFD Detection Reaction

CRISPR/Cas13a-LFD detection assays were performed with 45 nM LwCas13a, 22.5 nM crRNA, 125 nM RNA reporter, 1 μL of RNase inhibitor, 1 mM dNTP, 0.6 μL of T7 RNA polymerase, and 1 μL of RAA-amplified products. The sample with nuclease-free water, instead of the RAA reaction products, was set as a negative control. The reactions were incubated at a constant temperature of 37 °C for 40 min. Subsequently, 10 μL of CRISPR/Cas13a-LFD detection products were transferred into 90 μL of the detection buffer, and the strips were inserted. After 5–10 min incubation, specific bands were visualized and recorded using a digital camera.

To determine the limit of detection (LoD), the pUC57-p72 plasmid was serially diluted from 1 × 10^8^ copies/µL to 1 × 10^1^ copies/µL. Different dilutions of the plasmid as a template were amplified by RAA reaction, and then, the sensitivity of the CRISPR/Cas13a-LFD was analyzed with those reaction products.

In order to determine the specificity of this CRISPR/Cas13a-LFD assay, eight nucleic acid samples from porcine viruses including classical swine fever virus (CSFV), encephalomyocarditis virus (EMCV), Japanese encephalitis virus (JEV), porcine epidemic diarrhea virus (PEDV), pseudorabies virus (PRV), porcine circovirus 2 (PCV2), porcine circovirus 3 (PCV3), and porcine reproductive and respiratory syndrome (PRRSV) were tested.

To further verify the reliability of this method, 83 clinical samples were tested to analyze the coincidence rate between CRISPR/Cas13a-LFD and qPCR.

## 3. Results

### 3.1. The Detection of RAA-Amplified Products

**Figure 1:**
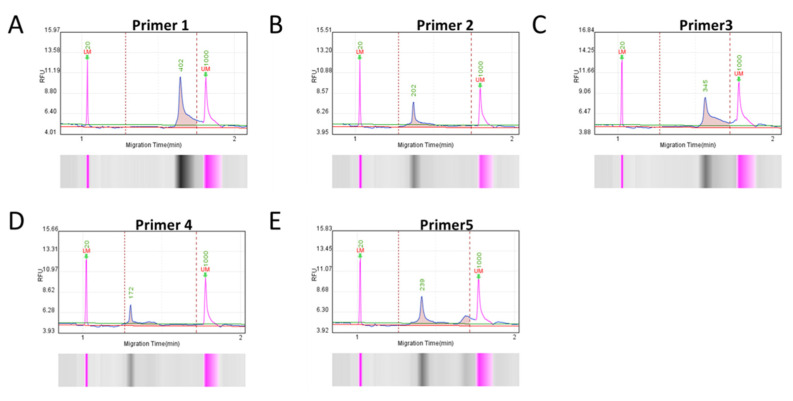
Capillary electrophoresis of RAA-amplified products, corresponding to five pairs of primers from (**A**–**E**). Five pairs of primers were used for RAA amplification; the product was analyzed by capillary electrophoresis.

To ensure specific and efficient amplification, five sets of RAA primers were designed based on the conserved nucleotide region of p72 gene. A Q-seq100 automatic nucleic acid analysis system was used to check the fragment size and concentration of RAA-amplified products to screen out qualified primers. The main peak fragment sizes of the corresponding RAA-amplified products were all close to the theoretical size (Figure 1), and the concentration of each product conformed to the requirement of the subsequent detection experiment (Table 3). Therefore, all primers were selected for further assessment in the subsequent experiments.

### 3.2. Establishment of the CRISPR/Cas13a-LFD

**Figure 2:**
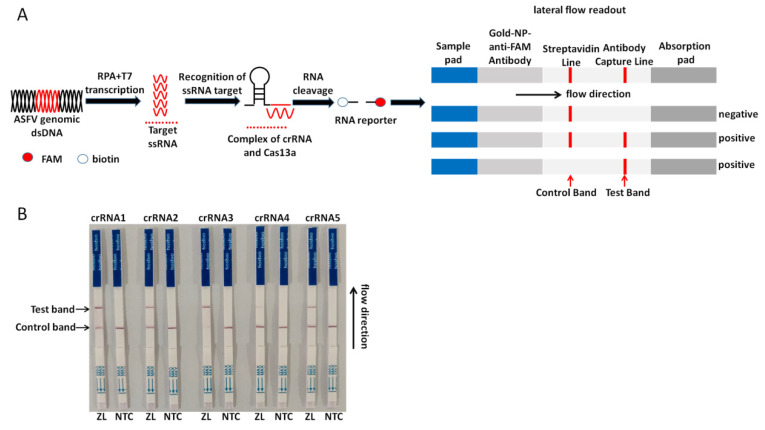
Establishment of the CRISPR/Cas13a-LFD. (**A**) Schematic diagram in the whole process of the CRISPR/CRISPR-associated 13 (Cas13a)-LFD detection. The P72 gene in the ASFV genome is amplified by RAA and then transcribed into RNA, which activates the Cas13a nuclease after being recognized by crRNA. The activated Cas13a nuclease then cuts off the reporter molecule, and the reporter molecule can appear as a band on the test strip. (**B**) Analysis of different crRNAs by CRISPR/Cas13a-LFD. ZL, pUC57-p72 standard plasmid. NTC, negative control.

For the on-site detection, we combined RAA, LwCas13a, and the lateral flow strip to establish the CRISPR/Cas13a-LFD assay. For the lateral flow detection, a FAM-RNA-biotin reporter was used. For the negative sample, a gold-NP-anti-FAM antibody was sufficiently conjugated with a FAM-RNA-biotin reporter, and the conjugate was intercepted by a biotin ligand at the control band. For the positive sample, the FAM-RNA-biotin reporter was cleaved, the conjugate of the gold-NP-anti-FAM antibody-FAM was accumulated at the test band, and accumulation was decreased at the control band. The schematic of the lateral flow detection is described in Figure 2A. Five Cas13a crRNAs targeting the RAA-amplified products of ASFV p72 were designed. The results are shown through strips; all the crRNAs were selected to perform the CRISPR/Cas13a-LFD assay (Figure 2B).

### 3.3. Sensitivity Test of the CRISPR/Cas13a-LFD

**Figure 3:**
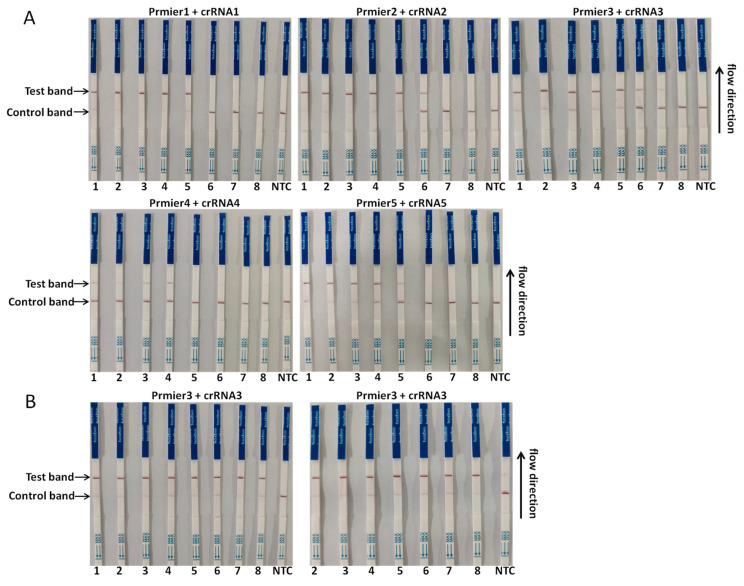
Sensitivity test of the CRISPR/Cas13a-LFD. A ten-fold serial dilution of pUC57-p72 was used as the detection template. (**A**) Sensitivity test of different primer and probe combinations; (**B**) sensitivity of the primer3 + crRNA3 combination after reaction system optimization. Detection reaction was performed independently total twice, with the detection limits of the following: 1:1 × 10^8^ copies/µL, 2:1 × 10^7^ copies/µL, 3:1 × 10^6^ copies/µL, 4:1 × 10^5^ copies/µL, 5:1 × 10^4^ copies/µL, 6:1 × 10^3^ copies/µL, 7:1 × 10^2^ copies/µL, 8:1 × 10^1^ copies/µL.

To optimize the performance of the assay, the sensitivity of the CRISPR/Cas13a-LFD based on different primer pairs and probe combinations was determined with a 10-fold serial diluted template at a concentration from 1 × 10^8^ copies/µL to 1 × 10^1^ copies/µL of the template. The combination of primer3 + crRNA3 had the highest sensitivity, of which the detection limit could reach 10^2^ copies/µL; primer2 + crRNA2 composition could reach the detection limit of 10^3^ copies/µL. The lower detection limit of primer1 + crRNA1 and primer5 + crRNA5 could reach 10^4^ copies/µL; the combination of primer4 + crRNA4 had a poor sensitivity, and the lower detection limit could only reach 10^5^ copies/µL (Figure 3A). To further improve the sensitivity of this assay, the primer3 + crRNA3 combination was selected for further optimization. The results indicated that when the amount of RAA-amplified product added was increased from 1 µL to 2 µL, the CRISPR/Cas13a-LFD was able to detect 1 × 10^1^ copies/µL of the DNA in the reaction, which was the ultimate LoD of this method (Figure 3B).

### 3.4. Specificity Test of the CRISPR/Cas13a-LFD

**Figure 4:**
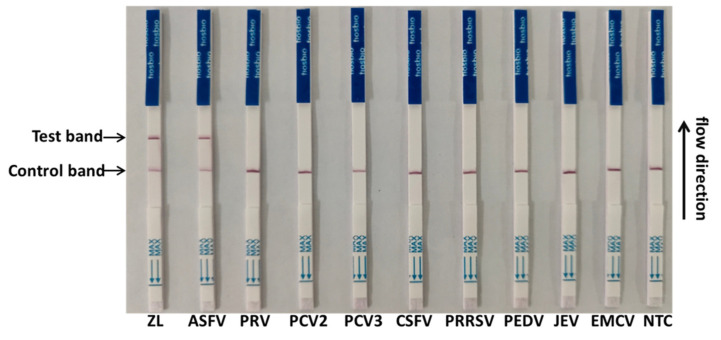
Specificity test of the CRISPR/Cas13a-LFD. Four DNA viruses (African swine fever virus (ASFV), pseudorabies virus (PRV), porcine circovirus 2 (PCV2), and PCV3) and five RNA viruses (classical swine fever virus (CSFV), porcine reproductive and respiratory syndrome (PRRSV), porcine epidemic diarrhea virus (PEDV), Japanese encephalitis virus (JEV), and encephalomyocarditis virus (EMCV)) were tested.

To evaluate the specificity of the CRISPR/Cas13a-LFD, the established assay was used for detection against other swine pathogens, including CSFV, PRRSV, EMCV, JEV, PCV2, PCV3, PEDV, and PRV. The primer3 + crRNA3 combination was selected for detection. The results showed that all the lateral flow strips of eight swine pathogens were negative bands, while the positive band was exclusively observed on the lateral flow strip of the ASFV (Figure 4). The results showed that the method has a high specificity for the detection of the ASFV.

### 3.5. Clinical Samples Detection by the CRISPR/Cas13a-LFD

**Figure 5:**
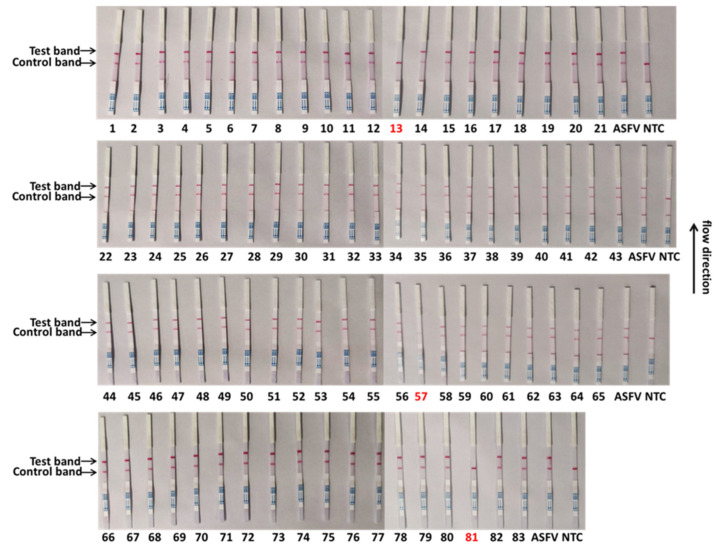
Clinical sample detection by the CRISPR/Cas13a-LFD. The samples marked with black numbers were ASFV-positive samples, and the samples marked with red numbers were negative samples. Eighty-three samples were used for testing.

**Figure 6:**
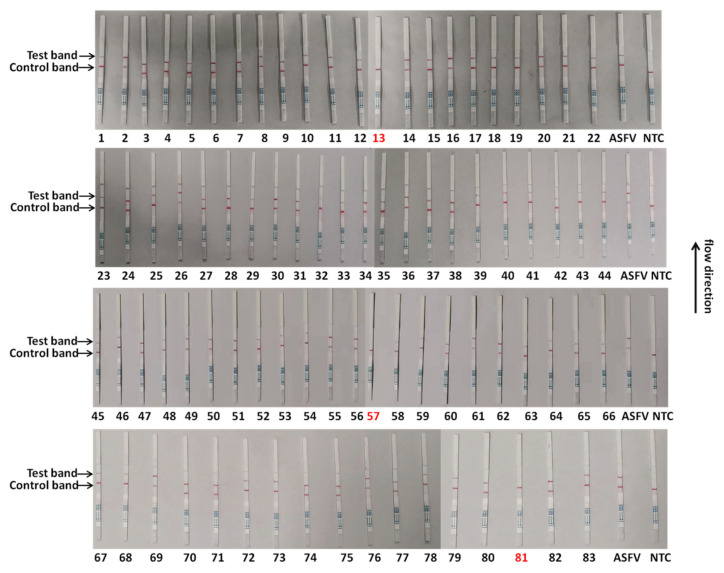
Repeated detection of clinical samples by the CRISPR/Cas13a-LFD. The samples marked with black numbers were ASFV-positive samples, and the samples marked with red numbers were negative. Eighty-three samples were used for testing.

To validate the reliability of the CRISPR/Cas13a-LFD in ASFV clinical sample detection, the CRISPR/Cas13a-LFD was performed to analyze 83 qPCR-tested tissue nucleic acid samples. As shown in Table 4, the CRISPR/Cas13a-LFD displayed the same results as the qPCR in clinical samples, indicating that the coincidence rate of these two methods could reach 100%. Eighty samples were detected as positive for the ASFV using this method, while three samples were detected as negative. The test results were consistent with the qPCR (Figure 5). To check the reproducibility of the method, we established, the ASFV detection of all 83 clinical samples was repeated independently. The results of the two tests were consistent, proving that the method is reliable and repeatable (Figure 6). These data showed that the CRISPR/Cas13a-LFD had accurate detection capabilities and can be used for on-site clinical ASFV detection without expensive equipment.

## 4. Discussion

Since 2018, the rapid outbreak of ASF in China has caused a serious social and economic impact, which is limiting the trade of swine products and affecting food security [8]. Due to the lack of effective vaccine and treatment, ASF control strategies largely depend on the rapid detection and slaughter of infected pigs. At present, the ASFV molecular diagnostic techniques mainly rely on OIE-recommended conventional PCR and real-time qPCR technique methods. Although these technologies have been widely used to detect this disease, they are still not suitable for rapid clinical testing due to expensive instruments and professional operating systems. Thus, the development of a rapid ASFV detection method is critical to limit the expansion of the disease at an early stage. In this study, we developed a rapid, sensitive and specific ASFV detection method based on CRISPR/Cas13a, which combines an RAA and a lateral flow strip. This method can be performed by a non-professional. The user only needs to add the required reagents to the nucleic acid sample to be tested, to complete the reaction in a constant-temperature water bath and then to use the test strip to complete the test. The reagents involved in the detection assay are workable for longer than six months when stored at −15 °C~−25 °C. The water bath and pipettes are the main equipment needed to conduct the assay, showing its potential applications for on-site ASFV detection.

Since the specificity of RAA–CRISPR depends on the number of mismatches in the crRNA target nucleotide region, we compared the sequences of different genotypes of ASFV p72 [27,28]. Based on the alignment results, five sets of RAA primers and the corresponding crRNA were designed in the conserved nucleotide region of the ASFV p72 gene. According to the preliminary detection results of five pairs of primer–probe combinations, we selected the most sensitive combination for optimization.

The lateral flow strip was used to facilitate the on-site diagnosis of the test results [29]. To prevent false-positive results and control production costs, the concentration of streptavidin in the test strip was optimized according to the amount of the RNA reporter used in a single reaction. For negative samples, the gold-NP-anti-FAM antibody was sufficiently conjugated with the RNA reporter, and the conjugate was intercepted by streptavidin at the control band, so only one band could be observed on the C line. For positive samples, the result depends on the cleavage efficiency of crRNA [30,31]. If the cleavage efficiency is high, the RNA reporter is completely cut, a high-strength band is observed at the T line of the band, and no band is observed at the C line. On the contrary, if the RNA reporter is not completely cleaved, bands can be observed at the T line and C line of the band, and the strength of the band depends on the cleavage efficiency.

The LoD of the CRISPR/Cas13a-LFD can reach 10^1^ copies/µL, and its sensitivity demonstrated its capability for detecting the ASFV. The CRISPR/Cas13a-LFD exhibited a similar sensitivity to the traditional qPCR method recommended in the OIE manual. Previous studies also reported an LFD method for PRRSV and ASFV with a lower level of sensitivity [32,33]. At the same time, the test showed no cross-reactivity with eight other swine viruses, proving its excellent specificity, which is very important in clinical testing. To verify the reliability of the CRISPR/Cas13a-LFD ASFV in clinical diagnosis, 80 cases of ASFV-positive specimens and three cases of ASFV-negative specimens verified by qPCR were analyzed, and the coincidence rate between the CRISPR/Cas13a-LFD and the qPCR was calculated. The results showed that the CRISPR/Cas13a-LFD was available for the on-site diagnosis of ASFV clinical cases with a coincidence rate of 100% for ASFV-positive and -negative samples.

Although real-time PCR is more sensitive, the CRISPR/Cas13a-LFD showed 100% agreement with a real-time PCR in detecting 83 clinical samples in this study. We speculate that this may be because the copy number of viral genomes in the clinical samples was higher than the LoD of the CRISPR/Cas13a-LFD. As genotype II of the ASFV is the predominant strain currently circulating in China, all the samples we collected were genotype II. Therefore, this method was only validated by clinical samples containing ASFV genotype II strains. There are at least 24 ASFV genotypes identified worldwide based on the nucleotide sequences of the variable 3′-end of the p72 gene [34]. The genetic variation between ASFV strains from different p72 genotypes was 9.4% at the nucleotide sequence level [35]. However, the specificity of RAA–CRISPR depends on the number of mismatches in the crRNA target region. In the present study, 17 p72 sequences covering genotypes I, II, III, IV, V, and X were used for the primer design. The highly conserved region among all six genotypes was selected for crRNA targets. There was no mismatch at the target regions of all the crRNAs on the ASFV P72 gene of genotypes I, II, III, IV, V, and X in the present method. In theory, it should have comparable performances for the detection of the genotypes I, II, III, IV, V, and X ASFV. However, additional samples infected with other genotypes of the ASFV or from other species such as wild boars need to be tested in the future to evaluate its potential application for monitoring the ASFV in the field.

In this study, the reaction time of the CRISPR/Cas13a-LFD can be completed within one hour. However, it did not consider the extraction time required for the nucleic acid sample preparation. This may become a limiting factor of our method in the field application. It would be helpful to develop a more appropriate and optimized extraction method to provide a basis for future on-site sample testing.

In conclusion, this study demonstrated that the CRISPR/Cas13a-LFD is a rapid, sensitive, specific, and portable method for ASFV diagnosis. The CRISPR/Cas13a-LFD has great potential in the field of ASFV detection and can be used as an effective way to monitor ASFV in time and prevent its occurrence and early spreading.

## Figures and Tables

**Table 1:** Sequences of the recombinase-aided amplification (RAA) primers.

Name		Sequence (5′-3′)
Primer 1	ASFV-F1	GAATGCGTACCGAAACTTGGTTTACTACTG
	ASFV-R1	CTTGTTTACCTGCTGTTTGGATATTGTGAG
Primer 2	ASFV-F2	GACGCAACGTATCTGGACATAAGACGTAATG
	ASFV-R2	CAAGCTTTATGGTGATAAAGCGCTCGCCGA
Primer 3	ASFV-F3	AAATCCTCATCAACACCGAGATTGGCACAA
	ASFV-R3	TTCAAAGCAAAGGTAATCATCATCGCACCC
Primer 4	ASFV-F4	CATCAATAACCTGTTTGTAACCCCTGAAAT
	ASFV-R4	TATTCAATGGGCCATTTAAGAGCAGACATT
Primer 5	ASFV-F5	TGCTAACGATGGGAAGGCCGACAAGATTAT
	ASFV-R5	TACCCGTATGCGGGCGTACTTTATTGTATT

**Table 2:** Sequences of clustered regularly interspaced short palindromic repeats RNAs (crRNAs).

Name	Sequence (5′-3′)
ASFV-crRNA1	ATCATTTTCATCGGTAAGAATAGGTTTG
ASFV-crRNA2	GTATTTAGGGGTTTGAGGTCCATTACAG
ASFV-crRNA3	TTATCGATAAGATTGATACCATGAGCAG
ASFV-crRNA4	GGTCACCTGCGTTTTATGGACACGTATC
ASFV-crRNA5	TTTCTTCGATTTGACTCAAAGTGGGTTC

**Table 3:** Amplified products use Q-sep100 detection results.

Primer Number	Peak Fragment Size (bp)	Theoretical Fragment Size (bp)	Product Concentration (ng/ μL)
Primer 1	402	396	3.71
Primer 2	202	212	2.58
Primer 3	345	349	3.79
Primer 4	172	181	2.15
Primer 5	239	243	3.55

**Table 4:** The coincidence rate between the CRISPR/Cas13a-LFD and real-time qPCR for the ASFV detection in clinical samples.

		Result (Positive/Negative)	
Sample Types	Number	CRISPR/Cas13a-LFD	qPCR
Blood	33	33/0	33/0
Nasopharyngeal swabs	25	25/0	25/0
Spleen	7	7/0	7/0
Liver	5	3/2	3/2
Lung	9	9/0	9/0
Kidney	4	3/1	3/1
In total	83	80/3	80/3

## Data Availability

All data generated and analyzed in this research are included in the article.

## Supplementary Materials

The following supporting information can be downloaded at: https://www.mdpi.com/article/10.3390/v14020179/s1, File S1: The detailed sequence of the p72 (GenBank: MK333180.1) fragment used in this study is supplemented as follows.
