# Supplementary material for: Rapid Detection of Genotype II African Swine Fever Virus Using CRISPR Cas13a-Based Lateral Flow Strip
Source: Viruses. 2022 Jan 18;14(2):179. doi: 10.3390/v14020179 (PMC8879322; doi:10.3390/v14020179)
Supplement: Supplementary file 1 [file viruses-14-00179-s001.zip › viruses-1507374-supplementary.pdf]

The detailed sequence of the p72 (GenBank:MK333180.1) fragment used in this study is supplemented as follows.

ATGGCATCAGGAGGAGCTTTTTGTCTTATTGCTAACGATGGGAAGGCCGACAAGATTATA
TTGGCCCAAGACTTGCTGAATAGCAGGATCTCTAACATTAAAAATGTGAACAAAAGTTAT
GGGAAACCCGATCCCGAACCCACTTTGAGTCAAATCGAAGAAACACATTTGGTGCATTTT
AATGCGCATTTTAAGCCTTATGTTCCAGTAGGGTTTGAATACAATAAAGTACGCCCGCAT
ACGGGTACCCCCACCTTGGGAAACAAGCTTACCTTTGGTATTCCCCAGTACGGAGACTTT
TTCCATGATATGGTGGGCCATCATATATTGGGTGCATGTCATTCATCCTGGCAGGATGCT
CCGATTCAGGGCACGTCCCAGATGGGGGCCCATGGGCAGCTTCAAACGTTTCCTCGCAAC
GGATATGACTGGGACAACCAAACACCCTTAGAGGGCGCCGTTTACACGCTTGTAGATCCT
TTTGGAAGACCCATTGTACCCGGCACAAAGAATGCGTACCGAAACTTGGTTTACTACTGC
GAATACCCCGGAGAACGACTTTATGAAAACGTAAGATTCGATGTAAATGGAAATTCCCTA
GACGAATATAGTTCGGATGTCACAACGCTTGTGCGCAAATTTTGCATCCCAGGGGATAAA
ATGACTGGATATAAGCACTTGGTTGGCCAGGAGGTATCGGTGGAGGGAACCAGTGGCCCT
CTCCTATGCAACATTCATGATTTGCACAAGCCGCACCAAAGCAAACCTATTCTTACCGAT
GAAAATGATACGCAGCGAACGTGTAGCCATACCAACCCGAAATTTCTTTCACAGCATTTT
CCCGAGAACTCTCACAATATCCAAACAGCAGGTAAACAAGATATTACTCCTATCACGGAC
GCAACGTATCTGGACATAAGACGTAATGTTCATTACAGCTGTAATGGACCTCAAACCCCT
AAATACTATCAGCCCCCTCTTGCGCTCTGGATTAAGTTGCGCTTTTGGTTTAATGAGAAC
GTGAACCTTGCTATTCCCTCAGTATCCATTCCCTTCGGCGAGCGCTTTATCACCATAAAG
CTTGCATCGCAAAAGGATTTGGTGAATGAATTTCCTGGACTTTTTGTACGCCAGTCACGT
TTTATAGCTGGACGCCCCAGTAGACGCAATATACGCTTTAAACCATGGTTTATCCCAGGA
GTCATTAATGAAATCTCGCTCACGAATAATGAACTTTACATCAATAACCTGTTTGTAACC
CCTGAAATACACAACCTTTTTGTAAAACGCGTTCGCTTTTCGCTGATACGTGTCCATAAA
ACGCAGGTGACCCACACCAACAATAACCACCACGATGAAAAACTAATGTCTGCTCTTAAA
TGGCCCATTGAATATATGTTTATAGGATTAAAACCTACCTGGAACATCTCCGATCAAAAT
CCTCATCAACACCGAGATTGGCACAAGTTCGGACATGTTGTTAACGCCATTATGCAGCCC
ACTCACCACGCAGAGATAAGCTTTCAGGATAGAGATACAGCTCTTCCAGACGCATGTTCA
TCTATATCTGATATTAGCCCCGTTACGTATCCGATCACATTACCTATTATTAAAAACATT

TCCGTAACTGCTCATGGTATCAATCTTATCGATAAATTTCCATCAAAGTTCTGCAGCTCT

TACATACCCTTCCACTACGGAGGCAATGCGATTAAAACCCCCGATGATCCGGGTGCGATG

ATGATTACCTTTGCTTTGAAGCCACGGGAGGAATACCAACCCAGTGGTCATATTAACGTA

TCCAGAGCAAGAGAATTTTATATTAGTTGGGACACGGATTACGTGGGGTCTATCACTACG

GCTGATCTTGTGGTATCGGCATCTGCTATTAACTTTCTTCTTCTTCAGAACGGTTCAGCT

GTGCTGCGTTACAGTACCTAA
